# Effect of Renal and Left Ventricular Function on Serial Pulmonary Arterial Pressure Changes after Device Closure of Atrial Septal Defect

**DOI:** 10.1155/2021/8846656

**Published:** 2021-01-17

**Authors:** Chul Shin, Young Won Yoon, In-Soo Kim, Jong-Youn Kim, Pil-Ki Min, Byoung Kwon Lee, Bum-Kee Hong, Se-Joong Rim, Hyuck Moon Kwon, Eui-Young Choi

**Affiliations:** Division of Cardiology, Heart Center, Gangnam Severance Hospital, Yonsei University College of Medicine, Seoul, Republic of Korea

## Abstract

**Background:**

The age of candidates for device closure of atrial septal defect (ASD) has been increasing. Thus, concerns exist about dyspnea aggravation or atrial fibrillation development after device closure due to augmentation of left ventricular (LV) and left atrial (LA) preload. This study aimed to examine patterns and determinants of serial pulmonary arterial pressure and left ventricular filling pressure changes after device closure of ASD.

**Methods:**

Among the 86 consecutive patients who underwent percutaneous device closure of ASD, those with end-stage renal disease or those without pre- or postprocedural Doppler data were excluded. The clinical, transesophageal, and transthoracic echocardiographic findings of 78 patients were collected at baseline, one-day postprocedure, and one-year follow-up.

**Results:**

The mean age of study patients was 49.8 ± 15.0 years, and the average maximal defect diameter and device size were 20.2 ± 6.0 mm and 23.8 ± 6.4 mm. Four patients (5.6%) underwent new-onset atrial fibrillation, and five patients (6.4%) took diuretics within one-year after closure. Some patients (*n* = 21; 27%) exhibited paradoxically increased tricuspid regurgitant velocity (TRV) one-day postprocedure; they also were older with lower e', glomerular filtration rate, and LV ejection fraction and a higher LA volume index. However, even in these patients, TRV deceased below baseline levels one-year later. Both E/e' and LA volume index significantly increased immediately after device closure, but all decreased one-year later. Larger defect size and higher TRV were significantly correlated with immediate E/e' elevation.

**Conclusion:**

In older, renal, diastolic, and systolic dysfunctional patients with larger LA and scheduled for larger device implantation, peri-interventional preload reduction therapy would be beneficial.

## 1. Introduction

The age of candidates for device closure of atrial septal defect (ASD) has been increasing. Especially in older patients, concerns exist about dyspnea aggravation [1] or atrial fibrillation development after device closure due to the augmentation of left ventricular (LV) and left atrial (LA) preload [2, 3]. Although recent studies showed that the long-term outcome of device closure is quite good even in older patients, vulnerable patients must be identified to prepare them for periprocedural preload reduction management. Thus, in this study, we sought to examine serial hemodynamic changes, including pulmonary arterial systolic pressure, LV filling pressure, stroke volume, and LA volume, after device closure of ASD. In addition, factors contributing to adverse hemodynamic changes from immediately after the procedure to one-year follow-up were determined.

## 2. Materials and Methods

### 2.1. Study Population and Clinical Follow-Up

**Figure 1:**
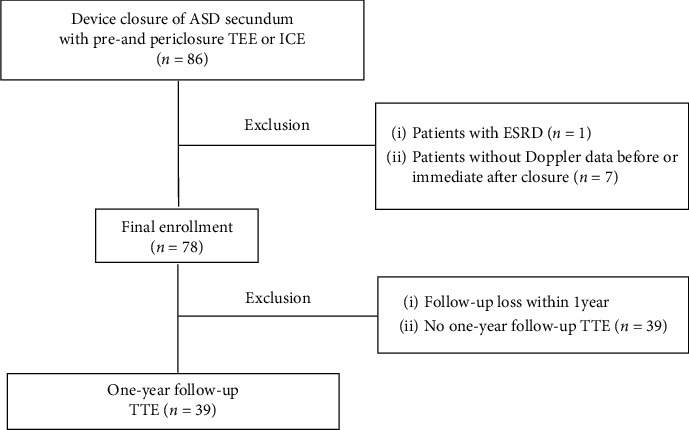
Schematic diagram of study patient selection process. ASD, atrial septal defect; ESRD, end-stage renal disease; TTE, transthoracic echocardiography; TEE, transesophageal echocardiography; ICE, intracardiac echocardiography.

The database of a single center of patients who underwent percutaneous device closure of ASD was analyzed. Patients underwent transthoracic echocardiography (TTE) and transesophageal echocardiography (TEE) before the device closure procedure. Among the 86 consecutive patients who underwent percutaneous device closure of ASD, patients with end-stage renal disease or patients without pre- or postprocedural Doppler data were excluded. In total, the clinical, transesophageal, and transthoracic echocardiographic findings of 78 patients were collected at baseline, one day (immediately) after the procedure, and one-year follow-up (Figure 1). All patients underwent blood chemistry and cell blood count analysis. Estimated glomerular filtration rate (eGFR) was calculated using the Chronic Kidney Disease Epidemiology Collaboration creatinine equation [4]. Clinical characteristics, medical history, laboratory findings, medications, and echocardiographic data were collected and used for analysis. The study protocol was approved by the institutional review board of Gangnam Severance hospital (3-2020-0026), and the need for written informed consent was waived due to the study's retrospective design.

### 2.2. Echocardiography at Baseline and after Device Closure

Each patient underwent a complete standard TTE. TTE findings were collected at baseline, one-day (immediately) postclosure, and one-year follow-up. LV dimensions and septal and posterior wall thickness were measured at end-diastole and end-systole in the two-dimensional (2D) parasternal long- or short-axis views. LV ejection fraction was calculated using the modified Quinones' method [5]. LV mass was measured by Devereux's methods as recommended by the American Society of Echocardiography [5]. LA volume was measured using the prolate ellipsoidal method at the point of LV end-systole at maximum LA size. From the apical window, a 1 mm pulsed Doppler sample volume was placed at the mitral valve tip, and mitral flow velocities from 5–10 cardiac cycles were recorded. Peak early (E) and late (A) mitral inflow velocities were also measured. Mitral annular velocity was measured by tissue Doppler imaging using the pulsed-wave Doppler mode. The filter was set to exclude high-frequency signals, and the Nyquist limit was adjusted to a range of 15–20 cm/s. Gain and sample volume were minimized to allow for a clear tissue signal with minimal background noise. Systolic (S') and early (e') and late diastolic velocities of the mitral annulus were measured from the apical four-chamber view with a sample volume (2–5 mm) placed at the septal corner of the mitral annulus. Peak velocity of tricuspid regurgitation was measured. Pulmonary arterial systolic pressure (PASP) was calculated as follows: 4 × tricuspid regurgitant velocity (TRV)^2^ + right atrial pressure, where right atrial pressure was estimated according to inferior vena cava diameter and its respiratory variations [6]. E/e' divided by stroke volume was defined as LV end-diastolic elastance index [7]. During TEE, multiplane 2D and zoomed 3D images were acquired, and both long- and short-axis diameters of the ASD were measured. Area was calculated as long-axis diameter multiplied by short-axis diameter and 3.14. In multiple defects, the sum of all diameters or areas was used. The margins to the aorta, posterior wall, superior vena cava, and inferior vena cava rims were also evaluated.

### 2.3. Device Implantation and Periprocedural Imaging

Transcatheter ASD closure was performed, as described previously [8], using various types of septal occluders (Amplatzer/Cocoon/Figullar Flex II/Gore-Helix/Occlutech). Before the procedure, the pulmonary-to-systemic blood flow ratio and pulmonary artery pressure were evaluated using cardiac catheterization. During the procedure, 3D-TEE or intracardiac echocardiography (ICE) were performed to guide accurate device implantation and ensure successful device closure. After the procedure, all patients received 100 mg/day aspirin for at least 6 months and 75 mg clopidogrel for 3 months. Other medications, such as diuretics and antihypertensive medications, were continued.

### 2.4. Statistical Analysis

Clinical characteristics and echocardiographic parameters are presented as the means ± standard deviation for continuous variables and the numbers (percentage) for categorical variables. Correlation analysis was performed between continuous variables using the Pearson correlation coefficient. Comparisons of clinical and echocardiographic findings between two groups were performed using the independent *t*-test. Nonparametric comparisons of two groups were done by Mann–Whitney *U* test. Serial changes in echocardiographic parameters were assessed using repeated ANOVA or the paired *t*-test. Variables with *P* values less than 0.05 in univariate analysis were included in the multivariable linear or logistic regression analysis. All the analyses were performed using SPSS (version 25.0, IBM, USA), and *P* values less than 0.05 were considered significant.

## 3. Results

### 3.1. Baseline Characteristics, Echocardiographic Parameters, and Hemodynamic Findings

The mean age of enrolled patients was 49.8 ± 15.0 (range, 16–77) years, and 51 (65%) were female. The average body mass index was 22.5 ± 3.0 kg/m^2^, and the average eGFR was 99.4 ± 20.0 mL/min. Mean LV ejection fraction and LV mass index were 66.1 ± 6.4% and 63.3 ± 16.3 g/m^2^, respectively. Among them, 4 patients had more than one defects. Measured average defect maximal diameter was 20.2 ± 6.0 mm, and defect area was 2.57 ± 1.52 cm^2^ on preprocedural TEE. The calculated Qp/Qs ratio during right side catheterization before device implantation was 2.52 ± 0.85. Sixty-four patients underwent TEE, and 14 patients underwent ICE for periprocedural guidance. The average size of the septal occluder was 23.8 ± 6.4 mm. Baseline characteristics are described in Table 1.

### 3.2. Clinical Problems after Device Closure

Six patients had history of persistent atrial fibrillation or documented paroxysmal atrial fibrillation. Among the rest (*n* = 72), four patients (4/72, 5.6%) underwent newly developed or detected atrial fibrillation within one year after device closure. Although it is not statistically significant due to small number of the patients, the patients with new-onset atrial fibrillation after closure had tendency of older age, lower eGFR, e', and LV ejection fraction, and larger defect size, LA volume index, and E/e. We could not find a case with development of significant pulmonary edema; however, five patients underwent taking diuretics after device closure. They had higher prevalence of atrial fibrillation, E/e', and lower eGFR, tendency of older age, lower eGFR, e', and higher LA volume index and E/e' without significance (Table 2).

### 3.3. Serial Changes in LV Filling Pressure, LA Volume, and Stroke Volume

**Figure 2:**
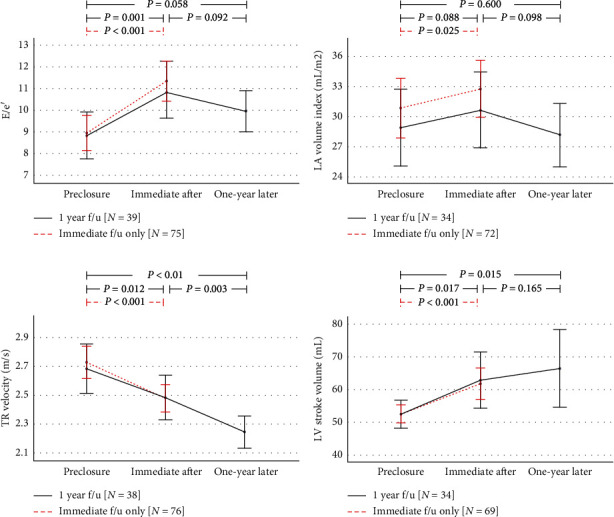
Serial changes in E/e' (a), left atrial (LA) volume index (b), tricuspid regurgitant (TR) velocity (c), and left ventricular (LV) stroke volume (d) from preclosure to one-year follow-up (*n* = 39). Blue dotted line represents comparisons between preclosure state and immediately after (one-day after closure) follow-up only (*n* = 78). Bars represent 95% confidence intervals.

At baseline, E/e' and LA volume index were 9.0 ± 3.5 and 30.5 ± 12.5 mL/m^2^, respectively. One day after the procedure, these values significantly increased to 11.4 ± 4.0 and 32.5 ± 12.1 mL/m^2^, respectively, due to increased LV filling from occlusion of a left-to-right shunt. LV stroke volume increased from 52.1 ± 11.2 mL to 61.5 ± 19.4 mL the day after the procedure. At one-year follow-up, E/e' and LA volume index decreased by 9.8 ± 3.0 and 27.8 ± 8.6 mL/m^2^, which was accompanied by a further increase in LV stroke volume (Figure 2). Immediate E/e' changes after device closure (ΔE/e'-immediate) were significantly correlated with immediate LA volume index change (*r* = 0.424; *P* < 0.001). ASD area, maximal defect diameter, and implanted device diameter were significantly correlated with ΔE/e'-immediate but not with age. Higher TRV and lower baseline E/e' were correlated with ΔE/e'-immediate (Table 3). In multivariate analysis, TRV and baseline E/e' were related to ΔE/e'-immediate.

### 3.4. Serial Changes in Pulmonary Arterial Pressure after Device Closure

**Figure 3:**
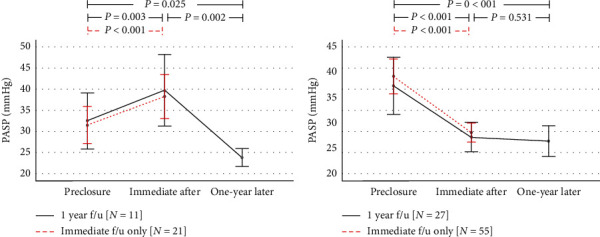
Serial changes in pulmonary arterial systolic pressure (PASP) from preclosure stage to one-year follow-up by immediate PASP response group: (a) immediately increased PASP group; (b) immediately decreased PASP group. Blue dotted line represents comparisons between preclosure state and immediately after (one-day after closure) follow-up only. Bars represent 95% confidence intervals.

**Figure 4:**
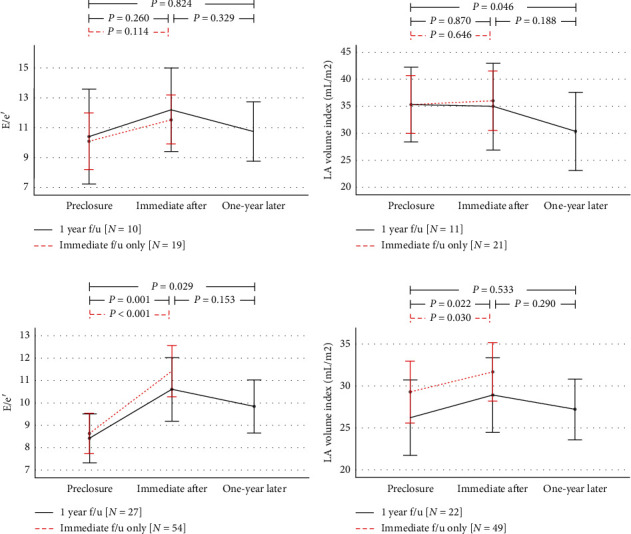
Serial changes in E/e', left atrial (LA) volume index, tricuspid regurgitant (TR) velocity, and left ventricular (LV) stroke volume from preclosure stage to one-year follow-up in (a, b) immediately increased PASP group and (c, d) immediately decreased PASP group. Blue dotted line represents comparisons between preclosure state and immediately after (one-day after closure) follow-up only. Bars represent 95% confidence intervals.

Of the 78 patients enrolled in the study, in two patients, pre- or post-TRV were not measurable and 39 underwent TTE at one-year follow-up. The average PASP decreased approximately −6.15 ± 11.6 mmHg immediately after closure, but some patients (*n* = 21; 28%) paradoxically exhibited increase in PASP. Patients with immediately increased PASP were older, with lower eGFR, LV ejection fraction, and e' and higher LA volume index (Table 4). In multivariate analysis for increased PASP-immediate, LV ejection fraction was significantly related to PASP increase immediately after closure. When serially followed, these patients also showed serially decreased PASP one-year later below the baseline level, similar to patients with initially decreased PASP (Figure 3). In patients with immediately increased PASP, accompanying increase in LA size was significantly blunted compared to decreased PASP group (Figure 4).

## 4. Discussion

According to our study results, we found some patients underwent new-onset atrial fibrillation and took diuretics within one-year after device closure. In addition, some patients who underwent device closure of ASD exhibited immediate PASP elevation even after transpulmonary arterial flow was reduced by blocking left-to-right shunt flow. This finding may be from immediately elevated pulmonary capillary wedge pressure due to increased LV filling flow to the noncompliant left ventricle or volume overload to the noncompliant left atrium (perhaps from not only the muscular part but also the device-covered noncompliant part) [9]. This speculation was supported by our findings that impaired LV relaxation (as represented by lower e') and systolic function, and higher LA volume index was found in the initial increased PASP group. This finding was more predominant in older patients with lower eGFR, suggesting that these patients need peri-interventional preload reducing medication, especially those with impaired diastolic function and larger LA size. When elevated LV filling pressure measured by E/e' was seen after device closure, larger defect size was more related to LV filling pressure elevation. Thus, especially in patients scheduled to close a larger defect, physicians should be cautious to avoid postinterventional pulmonary edema or exertional dyspnea aggravation. When considering the poor correlation between delta PASP elevation and delta E/e' elevation after device closure, increased PASP would be contributed by not only elevated LV filling pressure [10] but also LA noncompliance, which is also contributed by the device itself in the LA septum. An immediate increase in E/e' was significantly correlated with increase in LA volume index. However, at one-year follow-up, decreased LV filling pressure was accompanied by decreased LA volume index. When examining serial changes at one-year follow-up, we found that all patients showed decreased PASP below the baseline level regardless of the immediate PASP response, even in the paradoxically increased PASP group. Thus, despite the concern of device closure in older patients with renal dysfunction, these findings support that long-term hemodynamic response would be favorable in older patients. Therefore, with optimal preload reduction and volume control during and immediately after the procedure, device closure of ASD can be recommended even in vulnerable patients [11].

This study has some limitations. Although we found a significant proportion of patients underwent paradoxically increase in PASP, especially in older patients, with renal dysfunction, impaired LV relaxation, and larger LA, the contribution of the device on the LA side could not be fully determined. We could only assume that, with larger LA and marginal compliance, adding a prosthesis may worsen LA compliance. Although we could not find any difference in device type, future studies are warranted to determine how to reduce worsening LA compliance when selecting device size, device type, and deployment method.

## 5. Conclusions

In older patients with impaired LV relaxation, systolic dysfunction, renal dysfunction, or larger LA size, PASP could paradoxically increase after device closure of ASD due to the immediate volume overload to a noncompliant left ventricle and left atrium. Therefore, periprocedural preload manipulation, such as diuretics, is recommended in patients with these risk factors who are scheduled to close a larger defect. However, after the immediate periprocedural period, TRV, LA volume index, and E/e' continuously decrease, suggesting the favorable effects of ASD closure even in these high-risk patients. The risk for significant symptoms of heart failure is small with due precautions as none of the patients in this cohort experienced any.

## Figures and Tables

**Table 1:** Baseline clinical characteristics and echocardiographic measurements in patients with ASD.

Variable (*n* = 78)	Parameter
Age, years	49.8 ± 15.0
Female, *n* (%)	51 (65)
Body mass index, kg/m^2^	22.4 ± 3.0
Systolic blood pressure, mmHg	116.5 ± 15.7
Blood urea nitrogen, mg/dL	15.1 ± 4.4
Creatinine, mg/dL	0.75 ± 0.16
eGFR, mL/min/1.73 m²	99.8 ± 18.8
Atrial fibrillation, *n* (%)	6 (8)
LV mass index, g/m^2^	63.3 ± 16.3
LA volume index, mL/m^2^	30.5 ± 12.5
LV ejection fraction, %	66.1 ± 6.4
Stroke volume, mL	52.1 ± 11.2
TRV, m/sec	2.73 ± 0.48
PASP, mmHg	37.0 ± 12.3
PVR, Wood unit	1.47 ± 0.31
e', cm/s	9.3 ± 3.2
E/e'	9.0 ± 3.5
More than single defects, *n* (%)	4 (5)
ASD maximal defect diameter, mm	20.2 ± 6.0
ASD defect area, cm^2^	2.57 ± 1.52
ASD device diameter, mm	23.8 ± 6.4
Qp/Qs ratio	2.52 ± 0.85
Procedure guidance by TEE/ICE	64/14
Amplatzer, Abbott/Cocoon, Vascular Innovations/Figulla Flex II, Occlutech/Helex, Gore	41/33/3/1

ASD, atrial septal defect; e', early diastolic septal mitral annular velocity; E/e', ratio of early mitral inflow velocity to e'; ICE, intracardiac echocardiography; LA, left atrial; LV, left ventricular; PASP, pulmonary arterial systolic pressure; PVR, pulmonary vascular resistance; TEE, transesophageal echocardiography; TRV, tricuspid regurgitant velocity.

**Table 2:** Comparisons between groups according to new-onset atrial fibrillation after device closure and diuretics use.

Variable	New-onset AF after closure (*n* = 4)	^*∗*^No AF (*n* = 68)	^†^ *P* value	Diuretics use (*n* = 5)	No diuretics use (*n* = 73)	^†^ *P* value
Age, years	60.0 ± 10.5	47.9 ± 14.9	0.113	58.8 ± 13.7	49.2 ± 15.0	0.124
Female, *n* (%)	2 (50)	45 (66)	0.509	3 (60)	48 (66)	0.794
Past history of AF, *n* (%)				4 (80)	2 (3)	<0.001
eGFR, mL/min/1.73 m²	90.0 ± 10.6	102.8 ± 17.4	0.085	80.8 ± 14.1	101.2 ± 18.4	0.018
Maximal defect diameter, mm	23.0 ± 5.9	20.4 ± 6.0	0.308	20.0 ± 4.7	20.2 ± 6.2	0.886
Defect area, mm^2^	298.1 ± 110.8	262.1 ± 155.8	0.433	264.9 ± 151.4	256.5 ± 153.5	0.975
Device diameter, mm	26.0 ± 3.7	24.0 ± 6.5	0.491	22.8 ± 4.6	23.9 ± 6.5	0.779
LV mass index, g/m^2^	65.9 ± 17.5	62.8 ± 16.5	0.525	55.6 ± 14.4	63.9 ± 16.4	0.295
LA volume index, mL/m^2^	35.8 ± 6.5	28.1 ± 9.3	0.065	39.4 ± 20.4	29.9 ± 11.7	0.205
LV ejection fraction, %	62.3 ± 4.3	66.3 ± 6.6	0.198	65.4 ± 5.3	66.2 ± 6.5	0.772
Stroke volume, mL	51.6 ± 9.4	53.1 ± 10.7	0.832	40.7 ± 14.9	53.0 ± 10.5	0.038
TRV, m/sec	2.63 ± 0.36	2.72 ± 0.49	0.765	2.89 ± 0.56	2.72 ± 0.48	0.004
PASP, mmHg	36.8 ± 4.8	36.3 ± 12.6	0.549	45.5 ± 12.7	36.4 ± 12.2	0.306
PVR, Wood unit	1.24 ± 0.43	1.45 ± 0.27	0.336	1.91 ± 0.41	1.44 ± 0.28	0.088
e', cm/s	8.8 ± 3.5	9.3 ± 3.3	0.787	8.9 ± 1.7	9.3 ± 3.3	0.974
E/e'	10.7 ± 6.9	8.5 ± 3.0	0.681	11.5 ± 3.1	8.8 ± 3.4	0.038

AF, atrial fibrillation; see abbreviations in Table 1. ^*∗*^Patients with pre-existing AF were excluded; ^†^Mann–Whitney test for nonparametric comparisons.

**Table 3:** Correlations of immediate E/e' changes after device closure of an ASD.

Variable	Pearson's correlation coefficient (*r* value)	*P* value
Age, years	0.039	0.742
Male	−0.118	0.331
Body mass index, m^2^	−0.168	0.123
Systolic blood pressure, mmHg	−0.042	0.738
Blood urea nitrogen, mg/dL	0.112	0.338
Creatinine, mg/dL	0.183	0.115
eGFR, mL/min/1.73 m²	−0.110	0.349
ASD maximal defect diameter, mm	0.248	0.032
ASD defect area, cm^2^	0.309	0.008
ASD device diameter, mm	0.248	0.033
Qp/Qs ratio	0.217	0.103
LV mass index, g/m^2^	0.080	0.519
LA volume index, mL/m^2^	−0.078	0.509
LV ejection fraction, %	0.191	0.103
Stroke volume, mL	−0.137	0.251
TRV, m/sec	0.332	0.004
Preclosure E/e'	−0.359	0.002
Preclosure Ed	−0.227	0.055
ΔLA volume index-immediate	0.424	<0.001
Immediate LV Ed	0.480	<0.001

ASD, atrial septal defect; e', early diastolic septal mitral annular velocity; Ed, end-diastolic elastance; E/e', ratio of early mitral inflow velocity to e'; eGFR, estimated glomerular filtration rate; LA, left atrial; LV, left ventricular; TRV, tricuspid regurgitant velocity.

**Table 4:** Comparison between patients with increased PASP and decreased PASP immediately after device closure of ASDs^*∗*^.

Variable	PASP decrease (*n* = 55)	PASP increase (*n* = 21)	*P* value
Age, years	47.7 ± 14.4	56.4 ± 15.2	0.022
Female, *n* (%)	36 (66)	14 (67)	0.921
Body surface area, m^2^	1.62 ± 0.14	1.57 ± 0.18	0.288
Body mass index, kg/m^2^	22.3 ± 2.9	22.3 ± 3.1	0.986
Systolic blood pressure, mmHg	116.7 ± 13.7	115.1 ± 19.7	0.027
Atrial fibrillation, *n* (%)	4 (7)	2 (10)	0.745
Blood urea nitrogen, mg/dL	14.4 ± 3.7	16.7 ± 5.8	0.046
Creatinine, mg/dL	0.74 ± 0.15	0.80 ± 0.20	0.168
eGFR, mL/min/1.73 m²	102.6 ± 17.4	91.3 ± 20.3	0.018
Maximal defect diameter, mm	20.1 ± 6.2	21.1 ± 5.7	0.522
Defect area, mm^2^	253.0 ± 144.1	263.5 ± 156.4	0.794
Device diameter, mm	23.6 ± 6.5	25.0 ± 5.9	0.411
Qp/Qs ratio	2.46 ± 0.82	2.79 ± 0.95	0.214
LV mass index, g/m^2^	62.6 ± 14.1	64.4 ± 21.3	0.673
LA volume index, mL/m^2^	29.0 ± 12.5	35.4 ± 11.7	0.046
LV ejection fraction, %	67.4 ± 5.9	63.4 ± 7.0	0.015
Stroke volume, mL	52.4 ± 11.2	51.9 ± 11.7	0.869
TRV, m/sec	2.83 ± 0.46	2.48 ± 0.45	0.004
PASP, mmHg	39.2 ± 12.7	31.6 ± 9.6	0.016
PVR, Wood unit	1.49 ± 0.27	1.42 ± 0.42	0.425
e', cm/s	9.8 ± 3.0	7.7 ± 3.4	0.013
E/e'	8.65 ± 3.29	10.10 ± 3.94	0.120
Amplatzer, Abbott/Cocoon, Vascular Innovations/Figulla Flex II, Occlutech/Helex, Gore	29/23/3/0	10/10/0/1	0.266

^*∗*^In two patients, pre- or post-TRV were not measurable. ASD, atrial septal defect; e', early diastolic septal mitral annular velocity; E/e', ratio of early mitral inflow velocity to e'; eGFR, estimated glomerular filtration rate; LA, left atrial; LV, left ventricular; PASP, pulmonary arterial systolic pressure; PVR, pulmonary vascular resistance; TRV, tricuspid regurgitant velocity.

## Data Availability

The datasets used and/or analyzed during the current study are available from the corresponding author on reasonable request.

## Abbreviations

ASD: Atrial septal defect
E: Peak early diastolic mitral inflow velocity
e': Peak early diastolic mitral annular velocity
eGFR: Estimated glomerular filtration rate
ICE: Intracardiac echocardiography
LA: Left atrial
LV: Left ventricular
LVOT: LV outflow tract
PASP: Pulmonary arterial systolic pressure
TEE: Transesophageal echocardiography
TTE: Transthoracic echocardiography
TRV: Tricuspid regurgitant velocity
2D: Two dimensional
3D: Three dimensional.
